# LAG‐3—An incompletely understood target in cancer therapy

**DOI:** 10.1096/fj.202401639R

**Published:** 2024-11-19

**Authors:** Judith Leitner, Katharina Aigner‐Radakovics, Peter Steinberger

**Affiliations:** ^1^ Division for Immune Receptors and T Cell Activation, Institute of Immunology, Center for Pathophysiology, Infectiology and Immunology Medical University of Vienna Vienna Austria

**Keywords:** immune checkpoints, immunotherapy, inhibitory receptors, LAG‐3, T cell regulation

## Abstract

LAG‐3 is a member of the immunoglobulin superfamily expressed on activated T cells, but also on other immune cells. It has significant homology to CD4. Both molecules have four extracellular Ig‐like domains with similar structural motifs but the sequence identity between LAG‐3 and CD4 is low. Furthermore, unlike CD4 LAG‐3 restrains T cell responses and antibodies targeting this receptor are emerging drugs in cancer immunotherapy. A combination of LAG‐3 and PD‐1 antibodies has already been approved for the treatment of metastatic melanoma. Despite this success, its biology is still not well understood. Here we summarize the current knowledge on expression, ligands, and function of LAG‐3. We point to the differences between LAG‐3 and other inhibitory immune checkpoints and describe obstacles to study the role of this receptor in T cell activation processes. Finally, we discuss future directions for scientific efforts to come to a more complete understanding of the biology of this eminent immune checkpoint.

## Graphical abstract

This review deals with the biology of LAG‐3 and points to gaps in our knowledge such as the role of alternative ligands and signal transduction via this receptor. Furthermore, we discuss potential off‐target effects of LAG‐3 antibodies and future directions to come to a more complete understanding of LAG‐3.

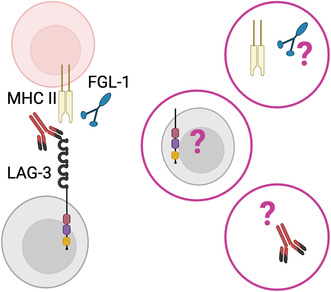

**Abbreviations**
APC: antigen presenting cell
CTLA‐4: cytotoxic T‐lymphocyte associated protein 4
FcγR: Fc‐gamma receptor
FGL1: fibrinogen‐like protein 1
ICI: immune checkpoint inhibitor
LAG‐3: lymphocyte‐activation gene‐3
PD‐1: programmed cell death protein 1
PD‐L1: programmed death‐ligand 1
TIM‐3: T cell immunoglobulin and mucin‐domain containing 3

## 1 INTRODUCTION

Immune checkpoint therapy has revolutionized cancer treatment, and antibodies targeting PD‐1, PD‐L1, and CTLA‐4 are successfully used to combat a wide variety of cancers.^1^, ^2^, ^3^, ^4^, ^5^, ^6^, ^7^, ^8^ In addition to these molecules, several other T cell coinhibitory receptors are considered promising targets for immune checkpoint inhibitor (ICI) therapy.^9^, ^10^, ^11^, ^12^, ^13^, ^14^ Among these, lymphocyte‐activation gene‐3 (LAG‐3; CD223) has recently become the third inhibitory pathway whose blockade was approved for clinical use. In March 2022, the US Food and Drug Administration (FDA) approved the LAG‐3 antibody relatlimab in combination with the PD‐1 antibody nivolumab for the treatment of advanced (unresectable stage III and stage IV) melanoma. This decision was largely based on the results of the RELATIVITY‐047 trial, a global, double blind, randomized phase 2–3 trial, where the combination of relatlimab and nivolumab was compared to nivolumab monotherapy, which is the standard of care for previously untreated metastatic or unresectable melanoma. 714 patients were enrolled, 355 of which received relatlimab and nivolumab, whereas 359 participants received nivolumab alone.^15^ The duration of progression free survival was significantly higher in the relatlimab‐nivolumab group (10.1 vs. 4.6 months). Likewise, the percentage of progression‐free survival was significantly higher in the group receiving both ICIs (47.7% vs. 36.0%). This improved survival is comparable to the one observed upon cotreatment with CTLA‐4 antibody ipilimumab and nivolumab (43.3%–49% vs. 37.7%) in an earlier study.^16^ Grade 3–4 treatment‐related adverse events were more frequently observed in the relatlimab‐nivolumab group (18.9%) versus 9.7% in the patients receiving only nivolumab but no new safety signals were observed. Furthermore, the safety profile of the relatlimab‐nivolumab combination appeared to be favorable compared to the combination of ipilimumab and nivolumab, but it should be noted that the two ICI combinations have not been compared in clinical trials. Overall, the results of the RELATIVITY‐047 trial demonstrate that co‐blockade of LAG‐3 and PD‐1 is an effective therapeutic strategy in melanoma and inaugurate LAG‐3 as the third T cell inhibitory pathway whose blockade is beneficial in the clinics. In addition to relatlimab, a large number of LAG‐3 targeting antibodies are currently evaluated in clinical and preclinical studies.^17^, ^18^, ^19^, ^20^ However, despite the longstanding efforts to introduce LAG‐3 targeting antibodies in the clinics and the recent success of relatlimab this pathway is still incompletely understood.^21^, ^22^ As we pointed out previously, there are considerable differences between PD‐1, which can be regarded as the classical T cell‐expressed immune checkpoint and inhibitory receptors such as CTLA‐4, TIM‐3, and LAG‐3. Moreover, each of these molecules has unique features, and there are considerable gaps in our knowledge regarding several aspects of the biology and function of these inhibitory receptors.^23^

In this review we focus on the biology of LAG‐3 and aim to summarize what is known regarding expression, ligands, and mode of action of this emerging target in cancer immunotherapy. Furthermore, we describe specific challenges in designing experiments that yield reliable information on the contribution of LAG‐3 to T cell activation processes. We discuss the limitations of experiments that rely on the use of blocking LAG‐3 antibodies and propose approaches that might potentially lead to better understanding of the role of LAG‐3 in T cell responses.

## 2 CHARACTERISTICS OF LAG‐3

LAG‐3, a type I transmembrane protein, is a member of the immunoglobulin superfamily with significant homology to CD4 and was first described by Triebel and coworkers in 1990.^24^ Both molecules have four extracellular Ig‐like domains (D1 to D4) with conserved structural motifs but the identity between LAG‐3 and CD4 is only 20% on the amino acid level.^24^, ^25^ LAG‐3 is largely absent on naïve CD4 and CD8 T cells, but, like other inhibitory receptors such as PD‐1, CTLA‐4, TIGIT, or TIM‐3, it is strongly upregulated upon activation. Furthermore, LAG‐3 is expressed in exhausted T cells, a trait it also shares with other inhibitory immune checkpoints.^26^, ^27^ Regulatory T cells express LAG‐3, which has been described to contribute to the suppressive function of these cells.^28^, ^29^, ^30^, ^31^ Apart from T cells, this receptor can be detected in other immune cells such as NK‐cells, B cells, and plasmacytoid dendritic cells (pDCs).^32^, ^33^, ^34^, ^35^ LAG‐3 has also been described to be expressed in non‐immune cells such as neurons.^36^ Murine LAG‐3 was reported to be cleaved between the D4 and the transmembrane domain by metalloproteases ADAM10 and ADAM17 presumably reducing the amount of surface expressed LAG‐3 and LAG‐3‐mediated inhibition.^37^ It is not known whether human surface LAG‐3 is also subject to proteolytical cleavage, and the functional role of soluble LAG‐3 is not well understood.^22^, ^38^, ^39^

## 3 THE IMPACT OF LAG‐3 DEFICIENCY ON T CELL FUNCTION

The results of numerous studies in mice point to an inhibitory function of LAG‐3 in immunity. Interestingly, in the initial study on LAG‐3 deficient mice Miyazaki et al. reported impaired NK cell function but no obvious defects in T cells.^40^ Subsequently, the same authors created CD4/LAG‐3 double deficient mice to test whether LAG‐3 might act as a substitute for CD4, which could explain the mild phenotype of mice lacking CD4 that was described in earlier studies.^41^, ^42^ However, these double deficient mice were found to be indistinguishable from CD4 deficient mice regarding lymphocyte populations and responses that are mediated by MHC class II molecules.^43^ In a more recent study, however, increased T cell expansion in response to stimulation was observed in LAG‐3 deficient mice.^44^ Furthermore, LAG‐3 was found to regulate T cell homeostasis as evidenced by significantly lower T cell numbers in wild‐type mice compared to mice lacking LAG‐3.^45^ In the context of LCMV infection, LAG‐3 deficient CD8 cells outcompeted their wild‐type counterpart and were sustained at higher levels.^46^ Accelerated diabetes with increased islet‐infiltration by autoreactive T cells was observed in LAG‐3‐deficient non‐obese diabetic (NOD) mice in independent studies.^47^, ^48^ Grebinoski et al. reported that intra‐islet CD8 T cells have a “restrained” exhausted phenotype and that selective deletion of cell‐bound LAG‐3 in CD8 T cells perturbed this functional state and accelerated diabetes onset in NOD mice.^49^ Furthermore, LAG‐3 function was also reported to ameliorate experimental autoimmune encephalomyelitis (EAE) and environmentally induced autoimmunity.^50^, ^51^ Okazaki et al. demonstrated that LAG‐3 deficiency alone did not induce autoimmunity in nonautoimmune‐prone mice strains but it induced myocarditis in BALB/c mice that additionally lacked PD‐1.^48^ Woo et al. also described strongly enhanced systemic autoimmunity in LAG‐3/PD‐1 double deficient mice and suggested synergistic regulation of self‐reactivity by these inhibitory receptors. Importantly, they also observed reduced tumor growth and enhanced survival in LAG‐3/PD‐1 double deficient mice in several models.^52^ LAG‐3 deficiency or blockade enhanced CD8 T cell accumulation and effector function towards tumors but also in a model for self‐tolerance.^53^ A recent study showed that CD8 cells lacking LAG‐3 and PD‐1 mediated improved tumor clearance. These cells were transcriptionally distinct and less prone to exhaustion. LAG‐3/PD‐1 double deficient CD8 cells were characterized by increased IFN‐γ production, and autocrine IFN‐γ signaling was required for tumor control by these cells.^54^

LAG‐3 is strongly expressed in Tregs and initial studies indicated that LAG‐3 might contribute to the suppressive function of these cells. Tregs from LAG‐3 deficient mice were reported to exhibit reduced regulatory activity and ectopic expression of LAG‐3 on CD4 T cells conferred on them suppressor activity towards effector cells.^28^ Furthermore, in adoptive transfer experiments Tregs from wild‐type but not from LAG‐3‐deficient mice reduced homeostatic expansion of CD4 T cells.^45^ Kim et al. showed that interleukin 27 (IL‐27) prevented EAE but failed to do so upon Treg depletion. Induced Tregs (iTregs) derived from wild‐type mice could reinstate the protective effects of IL‐27, whereas LAG‐3 deficient iTregs were ineffective.^55^ In another model, however, LAG‐3 deficient Tregs suppressed proliferation as efficiently as their LAG‐3 sufficient counterparts.^56^ Zhang et al. studied mice that selectively lacked LAG‐3 expression on Tregs in a murine model of diabetes. Unexpectedly, these mice exhibited significantly reduced diabetes incidence. LAG‐3 deficiency resulted in enhanced Treg proliferation and genes modulated by IL‐2/signal‐transducer and activator of transcription 5 (Stat5) signaling were upregulated. Eos, a coreceptor of Foxp3 was also upregulated and the authors provided evidence that suppression of Eos in LAG‐3 expressing Tregs compromises their proliferative capacity.^57^ Taken together, the studies on LAG‐3 deficient mice indicate that LAG‐3 suppresses expansion and potentially other functions in conventional T cells. The role of LAG‐3 in Treg appears to be complex and context‐dependent and warrants further studies.

## 4 LAG‐3 SIGNALING AND MODE OF ACTION

The ample interest in LAG‐3 as a clinical target is in stark contrast with the small body of studies addressing the mode of action of this receptor. Iouzalen et al. described LAG‐3 associated protein (LAP) as an intracellular interactor of LAG‐3, but they did not address the functional effects of this interaction.^58^ Moreover, there were no follow up studies on LAP function in the context of LAG‐3 and to our knowledge this interaction has not been confirmed by independent researchers. For almost two decades, a publication by Workman and colleagues was virtually the only study addressing the role of the cytoplasmic tail as well as a potential contribution of CD4 in LAG‐3 inhibition.^59^ However, in the last few years, several studies have revisited this topic and obtained divergent results.

**FIGURE 1:**
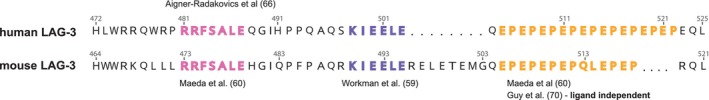
Motifs in the cytoplasmic tail of LAG‐3 implicated in mediating inhibitory effects.

LAG‐3 has a long cytoplasmic tail that harbors three conserved motifs, “RRFSALE,” “KIEELE” and the C‐terminal EP‐repeat motif (Figure 1). Workman et al. introduced LAG‐3 variants in the HEL‐peptide specific mouse hybridoma T cell line 3A9, which lacks endogenous expression of LAG‐3. Changes in the HEL‐peptide concentration required for the half maximum IL‐2 production (EC_50_‐value) were used as a readout for LAG‐3‐mediated inhibition in this study. Most importantly, the authors showed that the cytoplasmic tail of LAG‐3 is necessary for its inhibitory effects, because the full‐length, but not the tailless LAG‐3 required an increased peptide concentration for the activation of the HEL‐peptide specific hybridoma cells. They reported that the inhibitory activity of LAG‐3 depended on the KIEELE motif since the wild‐type LAG‐3, unlike the LAG‐3 variant lacking this motif, increased the HEL‐peptide EC_50_‐values. A LAG‐3 variant harboring a substitution of the lysine 468 also failed to inhibit, suggesting that this amino acid residue has a central role within this motif. Moreover, they reported that LAG‐3 inhibition was only occurring in the CD4^+^3A9 cell line and not in a CD4‐deficient subline (3A9.N49), which brought them to the conclusion that LAG‐3 inhibits T cell activation in a CD4‐dependent manner.^59^

Almost two decades later, Maeda et al. also used antigen‐dependent IL‐2 production in a mouse T cell hybridoma line (DO11.10) to study LAG‐3 function.^60^ They developed and used a highly effective blocking LAG‐3 antibody (TKB58) for their study. This gave them the possibility to compare the response of LAG‐3^+^DO11.10 cells in the presence and absence of LAG‐3 blockade, which is a more reliable method than comparing sublines with and without LAG‐3 expression as these might differ in their responsiveness in a LAG‐3 independent manner. The authors showed that the expression level of LAG‐3 strongly correlated with its inhibitory effect. They tested LAG‐3 variants with truncation in the cytoplasmic tail and in contrast to the Workman study, the deletion of the KIEELE motif did not significantly reduce LAG‐3 mediated inhibition in their model. Instead, they observed an important role of the membrane proximal (PR) sequences containing the RRFSALE motif (aa 473–479 of murine LAG‐3). Mutations of F475 and L478 strongly impaired the inhibitory capacity of LAG‐3, indicating that these two amino acid residues play an important role within this motif. Regarding the EP motif they observed that the deletion of the membrane‐distal EP motif did not significantly impair the inhibitory effect of LAG‐3 but on the other hand LAG‐3 variants lacking the PR‐motif retained around 50% activity, which could be indicative for a contribution of the EP motif to the ligand‐dependent inhibitory function of LAG‐3. Moreover, cells harboring a LAG‐3 variant with mutations in the RRFSALE motif and lacking the EP motif induced higher IL‐2 production than the control cells. This effect could be blocked with a LAG‐3 antibody, which led the authors to the conclusion that the remaining cytoplasmic tail of LAG‐3 harbored costimulatory activity.^60^

To clarify the impact of the different intracellular motifs on LAG‐3 function, our group aimed to compare all three motifs side by side in a standardized in vitro assay. We initially used a fluorescent transcriptional multiparameter reporter platform based on the human T cell line Jurkat. This cellular system can be used to concomitantly and independently read out the activity of three transcription factors that play major roles during T cell activation: NFAT, NF‐κB, and AP‐1.^61^ We have previously used fluorescent transcriptional reporter T cells to study major human coinhibitory pathways such as PD‐1, BTLA, NKG2A, CTLA‐4, TIM‐3, and TIGIT.^61^, ^62^, ^63^, ^64^, ^65^ Our results indicated that blocking LAG‐3 with antibody 25F7, the parent antibody of the therapeutic ICI relatlimab, enhanced NFAT, NF‐κB, and AP‐1 reporter gene induction in LAG‐3 expressing Jurkat cells stimulated by Staphylococcus aureus enterotoxin E (SEE) loaded RAJI.^66^ These experiments showed that LAG‐3 is functional in the Jurkat T cell line and inhibits the major transcription factors in T cell activation processes. We then set out to develop a LAG‐3 test system, in which T cell activation and LAG‐3 engagement are mediated by distinct ligands. To this end, we used T cell stimulators (TCS), which activate the T cell reporter cells via a membrane bound CD3‐single chain antibody (mb‐CD3‐scFv).^67^ In the stimulation assays, we used control TCS and TCS expressing the LAG‐3 ligand MHC class II to activate Jurkat reporter cells in the presence and absence of LAG‐3 engagement. Both stimulator cells induced comparable activation of control reporter cells, whereas the activation of LAG‐3 expressing NF‐κB::eGFP‐reporter cells was significantly reduced in the presence of MHC class II (HLA‐DR) expressing stimulator cells. Unlike previously used cell line‐based assay systems for LAG‐3, this reporter platform allowed to study LAG‐3 effects without relying on blocking LAG‐3 antibodies.^60^, ^68^, ^69^ Experiments with reporter cells expressing LAG‐3 variants revealed that the lack of the RRFSALE motif abrogated LAG‐3 mediated inhibition of eGFP reporter gene expression, while LAG‐3 variants without the KIEELE‐ or the EP‐motif retained full activity. Inhibition was abrogated upon mutation of the amino acid residues F483 or L486 within the RRFSALE motif.^66^ Of note, the corresponding amino acid residues in murine LAG‐3 (F475 and L478) were also described to be essential in murine LAG‐3 by Maeda et al.^60^ All in all, these results were in agreement with the data obtained by Maeda and colleagues with murine LAG‐3 and indicate that the RRFSALE motif has an important role in LAG‐3 mediated inhibition. It should be noted that Workman et al. did not assess LAG‐3 variants that lacked this motif in their study. Instead, they only tested a variant where the serine in the RRFSALE motif was mutated as a potential phosphorylation motif (LAG‐3.S454A).^59^ This mutation did not significantly impair the inhibitory capacity of LAG‐3, which is in line with our results that indicated that the corresponding serine (S484) is dispensable for inhibition via human LAG‐3.^66^

Recently, another report from the Workman group proposed that LAG‐3 also inhibits T cell activation in a ligand independent manner.^70^ CD4 and CD8 T cells from LAG‐3^ko^ mice were found to proliferate stronger than their counterparts from LAG‐3 sufficient animals. Moreover, they showed that LAG‐3 associates with the TCR/CD3 complex and disrupts the interaction of Lck with the coreceptors CD4 and CD8. This disruption occurs via the membrane distal cytoplasmic EP motif of LAG‐3. Divalent Zn‐cations mediate the association of Lck and CD4 or CD8 via a di‐cystine motif and excess of Zn^2+^ prevented the dissociation of Lck from CD4 via peptides representing the EP motif, implicating Zn‐ion sequestering by LAG‐3 in this process.^70^ The possibility of ligand independent inhibitory activity of LAG‐3 raises two important questions: (i) What is the contribution of ligand‐independent inhibition to the overall inhibitory effects of LAG‐3? (ii) Can ligand independent effects of LAG‐3 be targeted by immune checkpoint inhibitors?

Currently, it is not known how LAG‐3 connects with the intracellular signaling machinery to mediate T cell inhibition upon ligand engagement.^18^, ^21^, ^22^, ^71^ Consequently, it will be of utmost importance to identify molecules involved in LAG‐3 signal transduction to eventually dissect the downstream signaling pathway of this eminent immune checkpoint. Since the LAG‐3 pathway appears to be distinct from other inhibitory receptors, candidate molecules are not readily available for testing. Consequently, unbiased screening campaigns might be the most promising strategy to identify molecules that potentially have a role in LAG‐3 function. Proteomics‐based approaches that have recently been used to identify interactors for PD‐1, BTLA, or TIM‐3 might have great potential in this context.^72^, ^73^, ^74^ Another promising strategy is the screening of CRISPR‐Cas9 genome‐wide knock out libraries to identify genes required for LAG‐3 mediated inhibition of T cells.^75^

## 5 LAG‐3 LIGANDS AND THEIR CONTRIBUTION TO LAG‐3 INHIBITION

MHC class II was the first ligand for LAG‐3 to be identified.^76^ This interaction has been demonstrated to depend on the first and second Ig‐like domain of LAG‐3 and appears to involve the extra loop of the membrane distal D1 domain, which is absent in CD4, and amino acid residues located downstream of this loop.^71^, ^77^, ^78^ Importantly, LAG‐3 has been shown to transduce inhibitory signals into T cells upon engagement by MHC II.^60^, ^66^, ^77^, ^79^, ^80^ Consequently, MHC class II molecules are considered as the canonical ligands for LAG‐3, and the blocking capacity of LAG‐3 antibodies is usually gauged by their capacity to disrupt the interaction of LAG‐3 and MHC class II.

More recently, the soluble liver protein fibrinogen‐like protein‐1 (FGL1) has been identified as a LAG‐3 binding partner in a screening campaign, where soluble proteins were expressed as type I and type II transmembrane proteins. The authors reported an important role of FGL1 in LAG‐3 mediated inhibition in their in vivo model and proposed that FGL1 acts as a major functional ligand for LAG‐3.^81^ In part, these conclusions were based on results obtained with an antibody to murine LAG‐3, C9B7W, which according to the authors selectively blocked the FGL1‐LAG‐3 interaction. However, a more recent study demonstrated that this antibody also blocks the interaction of MHC class II molecules with LAG‐3.^82^ Wang et al. reported T cell inhibition via FGL1 and augmented T cell immunity in mice lacking FGL1. However, it has been shown that FGL1 binds to activated T cells in LAG‐3 deficient mice.^82^ Therefore, it is possible that FGL1 exerts inhibitory effects on T cells independently of LAG‐3. Silberstein et al. demonstrated that LAG‐3 dimerizes via its D2 domain and that LAG‐3 homodimerization is important for ligand binding. The LAG‐3 antibody C9B7W disrupted LAG‐3 dimerization thereby potently interfering with the binding of LAG‐3 to FGL1 as well as MHC class II.^83^ We could confirm a specific interaction between soluble and cell‐bound FGL1 and LAG‐3 but in our experimental systems, the capacity of FGL1 to induce inhibition via LAG‐3 was weak.^66^ Unlike other ligands for immune checkpoints, FGL1 is a soluble molecule. It has been shown to dimerize but it is currently not clear whether the engagement of dimeric FGL1 is sufficient to induce potent inhibitory signals via LAG‐3. Ming et al. also observed weak inhibitory effects of FGL1 in a Jurkat reporter model and demonstrated that FGL1 promotes LAG‐3 clustering at the cell surface.^84^ This effect was dose‐dependent and diminished at very high FGL1 concentrations. In the presence of 100 nM FGL1 LAG‐3 clustering was not higher than in the absence of FGL1 yet the inhibitory effect of FGL1 was maintained. Further studies are warranted to study the interrelation between the FGL1‐mediated LAG‐3 clustering and LAG‐3 mediated inhibition.

LAG‐3 is a heavily glycosylated protein and two lectins have been proposed to function as ligands for this receptor. In 2014, Xu and colleagues reported that LSECtin expressed on melanocytes mediates inhibition of T cell responses via LAG‐3.^85^ Shortly thereafter, the interaction of Galectin‐3 (Gal‐3) with LAG‐3 was described to suppress CD8 T cells and to inhibit pDC expansion.^86^ However, there were no follow‐up studies, and unlike for MHC class II or FGL‐1, the LAG‐3 binding site of these molecules has not been identified. Moreover, the interaction of LAG‐3 to these lectins has not been corroborated by independent studies. Binding studies performed in our laboratory did not confirm a specific interaction between LSECtin and LAG‐3 and neither the LSECtin nor Gal‐3 mediated LAG‐3 inhibition in our T cell reporter system.^66^

A‐Syn, a presynaptic neuronal protein, has been described as another ligand for LAG‐3.^36^ The authors proposed a role of a‐syn preformed fibrils (PFF) in the development of Parkinson's disease (PD) and reported that treatment with LAG‐3 antibodies blocked interaction with PFF and ameliorated PD pathology in a preclinical model.^36^ However, neither LAG‐3 expression in neuronal cells and tissues nor a specific interaction between LAG‐3 and PFF could be confirmed by a more recent study.^87^

Maruhashi et al. reported that LAG‐3 selectively interacts with cells expressing “stable peptide‐bound MHCII (pMHCII),” but not with cells expressing “unstable MHCII” molecules on their surfaces.^78^, ^82^ MHC class II molecules induced by class II transactivator (CIITA) expression mediated strong LAG‐3 binding and the authors proposed that CIITA‐induced accessory factors (such as the invariant chain and H2‐DM), but also peptides binding to MHC class II with high affinity, stabilized the surface MHC II molecules for LAG‐3 binding.^78^ Compared to CIITA induced MHC class II expression, we did not observe a weaker binding of LAG‐3 to HLA‐DRαβ ectopically expressed in “non‐professional antigen presenting cells (APC” such as Jurkat or K562 cells.^66^ One important difference between our study and the reports by Maruhashi et al. was that we studied human cells and human LAG‐3, whereas their studies were performed in murine systems. Therefore, it is possible that the discrepant results are due to species‐dependent differences between the conformational stability of MHC class II on cell surfaces.

Engagement of MHC class II molecules by a dimeric LAG‐3 fusion protein (LAG3‐Ig) has been reported to induce DC activation.^88^ Furthermore, the presence of LAG3‐Ig enhanced proliferation of antigen‐specific human T cells in vitro.^89^, ^90^ Vaccination studies carried out in mice demonstrated that LAG3‐Ig enhanced immune responses and slowed tumor growth. Based on these findings, hLAG3‐Ig protein (IMP321) was developed as an immune adjuvant and has since been tested in several clinical trials.^29^, ^90^, ^91^, ^92^, ^93^, ^94^ Yet, the mode of action of this drug and activating downstream pathways induced by MHC class II—LAG3‐Ig interaction remain poorly understood.

Taken together, MHC class II molecules are still the best characterized functional ligands for LAG‐3 and independent studies are required to gauge the contribution of alternative binding partners such as FGL1 to the LAG‐3‐mediated inhibition of T cell responses. Likewise, further work is necessary to establish a biological significance of “MHC class II stability” in human LAG‐3 function. Well‐defined tools such as monoclonal antibodies that reliably discriminate between “stable” and “unstable” MHC class II molecules on the surface of human cells would greatly facilitate such studies.

## 6 Future directions: Challenges in investigating LAG‐3 function

One important difficulty in studying LAG‐3 function is the fact that MHC class II, its major ligand, is also a ligand for the TCR‐CD3 complex. In many experimental settings, it is therefore not possible to study T cell activation processes in the absence or presence of LAG‐3 ligation, since the ligand also provides the activating signal (signal 1) to T cells. Frequently, investigations rely on the use of blocking LAG‐3 antibodies and attribute the enhancement of T cell activation upon LAG‐3 blockade to the inhibitory effects mediated by LAG‐3 engagement.

**FIGURE 2:**
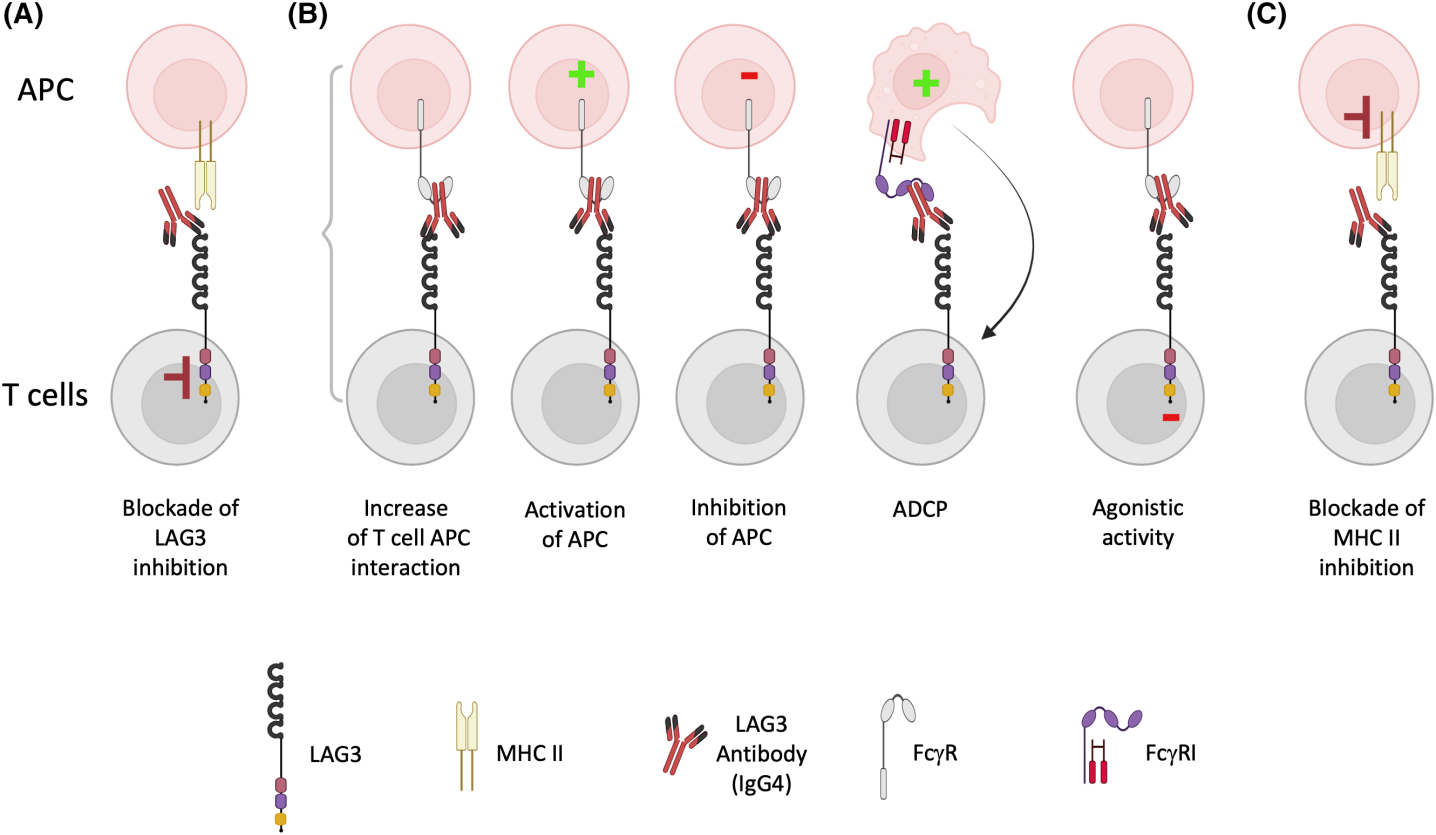
Potential effects of therapeutic LAG‐3 antibodies. Apart from their intended function as immune checkpoint inhibitors (A), LAG‐3 antibodies could potentially exert various FcγR mediated effects (B), and block the transduction of inhibitory signals generated upon the engagement of MHC class II by LAG‐3 (C). ADCP, Antibody dependent cellular phagocytosis. The figure was created using BioRender.

**FIGURE 3:**
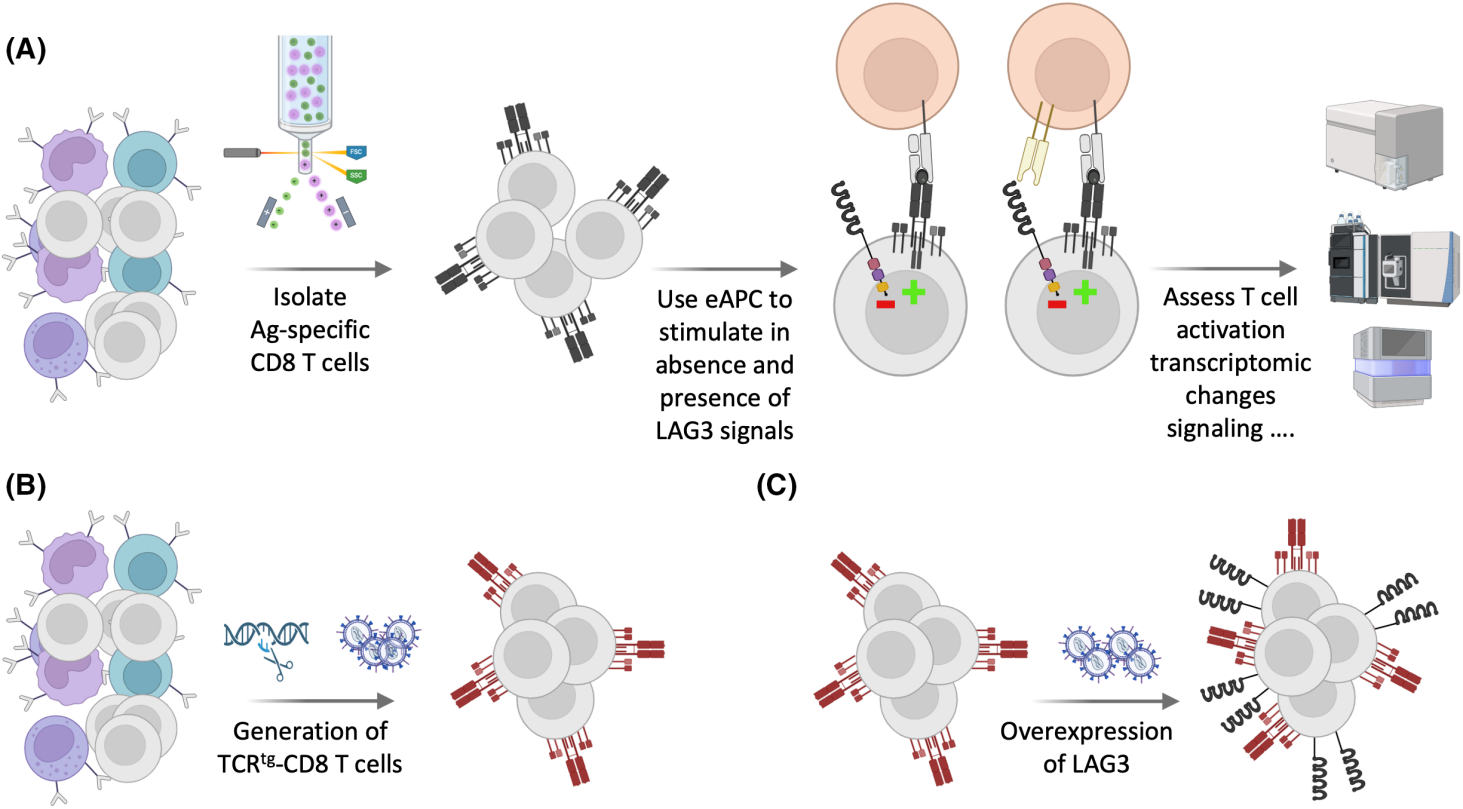
Studying LAG‐3 effects independent of blocking antibodies. (A) CD8 T cells recognizing common antigens (such as the NLVPMVATV peptide derived from the CMVpp65 antigen presented in the context of HLA‐A*0201) are isolated from human peripheral blood mononuclear cells (PBMCs) and stimulated by engineered antigen presenting cells (eAPC) expressing the LAG‐3 ligand MHC class II or not. This experimental setup can be used to study the impact of LAG‐3 engagement on various T cell activation processes such as upregulation of activation markers, proliferation, cytokine production but also on transcriptional reprograming (by bulk and single cell RNA sequencing) and signaling (e.g., by phosphoproteomics). (B) Alternatively, the endogenous TCR could be replaced to obtain large numbers of TCR‐transgenic (tg) CD8 T cells specific for the eAPC‐expressed peptide–MHC I complex. (C) Overexpression of LAG‐3 on antigen‐specific CD8 T cells should yield more pronounced effects of LAG‐3 and could thus potentially facilitate the assessment of LAG‐3 inhibition. The figure was created using BioRender.

Several studies have shown that LAG‐3 antibodies alone or in combination with PD‐1 blockade could enhance human T cell activation in vitro.^95^, ^96^, ^97^, ^98^, ^99^, ^100^ However, in many cases the effect of LAG‐3 antibodies was not very pronounced or only seen in combination with PD‐1 blockade. In some settings, LAG‐3 antibodies did not significantly augment cytokine production and proliferation of human T cells in vitro.^101^, ^102^, ^103^ Importantly, there is increasing evidence that blocking antibodies to immune checkpoints can exert stimulatory and inhibitory effects, which are independent from their capacity to block inhibitory signaling of their target receptors. Some of these effects depend on the binding of the therapeutic antibodies to activating or inhibitory Fc gamma receptors (FcγR). The therapeutic CTLA‐4 antibody ipilimumab, which is an unmutated IgG1 antibody, is a good example, since it was shown to mediate potent antibody‐dependent cellular cytotoxicity (ADCC) towards Tregs, which express high levels of CTLA‐4 in vitro, and potentially also towards CD4 effector T cells.^101^, ^104^ These results, as well as results obtained in preclinical models, suggest that cytotoxic effects towards Tregs contribute to the clinical effects of anti‐CTLA‐4 therapy.^105^, ^106^, ^107^, ^108^ The majority of ICIs targeting other immune checkpoints such as PD‐1, NKG2A, and LAG‐3 are IgG4 and should therefore not exert cytotoxic effects via FcγRIII(F158) or C1q, but there is increasing awareness that such antibodies can exert immunomodulatory effects that are distinct from their intended mode of action, namely the blockade of inhibitory signals generated by the targeted receptors.^109^, ^110^, ^111^, ^112^, ^113^, ^114^ IgG4 antibodies bind to FcγRI, FcγRIIa, FcγRIIb as well as to the V158‐variant of FcγRIII.^110^ This interaction could increase T cell responses by promoting the interaction of FcγR expressing APC with immune checkpoint expressing T cells. Furthermore, they could induce activating signaling in APC via FcγRI and FcγRIIa, which might promote their T cell stimulatory capacity. On the other hand, interactions with FcγRs have been shown to disrupt ICI‐mediated blockade by transferring antibodies to macrophages.^115^ Interaction of IgG4 with FcγRI can furthermore result in the elimination of immune checkpoint expressing T cells via antibody dependent cellular phagocytosis (ADCP).^110^, ^116^ FcγR interaction of immune checkpoint inhibitors might also induce inhibitory signaling, since crosslinking via Fc‐receptors could endow them with agonistic capacity towards their target.^117^, ^118^ Finally, engagement of APC‐expressed MHC class II by LAG‐3 expressing cells has been reported to transduce inhibitory signals into APC,^29^, ^119^ and consequently LAG‐3 antibodies could also act by disrupting this “reverse” inhibitory signaling via MHC class II. Potential effects of immune checkpoint inhibitors are summarized in Figure 2. We and others have observed that LAG‐3 antibodies can exert stimulatory effects, which are independent of LAG‐3 ligands.^66^, ^70^, ^120^ Especially for studies of LAG‐3 function in primary human T cells, it would therefore be helpful to establish experimental systems that allow studying of the LAG‐3 effects independent of blocking antibodies. This could potentially be attained by using an experimental system outlined in Figure 3. In the proposed setting, T cell stimulation in the presence or absence of LAG‐3 signaling could be achieved by using a matched pair of engineered antigen presenting cells (eAPC), which only differ in their expression of the LAG‐3 ligand MHC class II. The use of MHC I restricted CD8 T cells will prevent stimulation of alloreactive T cells by MHC class II molecules and assure that eAPC expressed MHC class II molecules exclusively function as LAG‐3 ligands in this system. Potentially, LAG‐3 mediated inhibition could be benchmarked by including PD‐L1 expressing eAPC. We have demonstrated that the inhibitory effects of the immune checkpoints PD‐1 and BTLA can be greatly enhanced by overexpressing them in primary T cells.^121^ Consequently, overexpression of LAG‐3 might be a viable strategy to enhance the inhibitory effects of LAG‐3 in primary T cells in case the inhibitory effects exerted by endogenous LAG‐3 would be weak. Using this approach, the effect of LAG‐3 inhibition could be comprehensively studied by analyzing standard T cell activation parameters such as proliferation, upregulation of activation markers and cytokine production. In addition, this experimental setting could also be used in “omics‐based studies” to gain insights into how LAG‐3 signals shape, for example, the transcriptome or phospho‐proteome of human T cells.

## 7 OUTLOOK

Many aspects of LAG‐3 biology are still enigmatic. Studies towards a better understanding of the function of this receptor might eventually translate into new and improved therapeutics. Additional information regarding the contribution of LAG‐3 ligands to its inhibitory function could potentially help to design improved LAG‐3 antibodies, which effectively block alternative ligands in addition to its canonical ligand MHC class II. The dissection of intracellular pathways that mediate the inhibitory effects of this receptor are a prerequisite for development of effective small compound inhibitors of LAG‐3 to enhance anti‐tumor responses in cancer patients.

## AUTHOR CONTRIBUTIONS

Judith Leitner and Peter Steinberger conducted the literature search and wrote the manuscript. Judith Leitner generated the figures. Katharina Aigner‐Radakovics participated in the literature search and contributed to writing the manuscript. All authors were involved in revising the manuscript and approved of the final version.

## FUNDING INFORMATION

This work was supported by a grant from the Austrian Science Fund (FWF P3241 to PS).

## DISCLOSURES

None of the authors has a conflict of interest regarding the manuscript.

## Data Availability

Data sharing is not applicable to this article as no datasets were generated or analyzed during the current study.
